# On the intergenerational transfer of ideas in aging and cancer research: from the hypothalamus according to V.M. Dilman to the mTOR protein complex according to M.V. Blagosklonny

**DOI:** 10.18632/aging.206338

**Published:** 2025-11-19

**Authors:** Aleksei G. Golubev

**Affiliations:** 1 N.N. Petrov National Medical Research Center of Oncology, Russia

**Keywords:** gerontology, history of science, hyperfunction, mTOR, hypothalamus, cancer, metabolism, immunity

## Abstract

Here, conceptual similarities between Mikhail Blagosklonny’s hyperfunction theory of aging and Vladimir Dilman’s elevation theory of aging are considered. Taken into account are the genealogical relations between the two eminent gerontologists, the commonality of their educational backgrounds, and my own experience of being affiliated with Dilman’s laboratory in the late 1970s and early 1980s. Based on this, I suggest some general considerations concerning the similarities and differences between the two men in their approach to understanding aging and longevity. Dilman’s scientific legacy is not as well recognized as it should be, partly due to bias in citation practices. The latter has become increasingly prevalent, partly because the enormously swelled body of literature on aging obscures much of what was pioneered decades ago.

*“Each generation stands on the shoulders of those who have gone before them…”*

*Steven Hawking*

Mikhail Vladimirovich Blagosklonny (1961–2024) was his father’s son. That would be nothing special, if not for the fact that his father was Vladimir Mikhailovich Dilman (1925–1994). In an insightful account of the history of aging research in Russia [1], it is declared that “Perhaps the last top achievements of gerontology in Soviet period were related to the names of two outstanding domestic scientists: Vladimir Mikhailovich Dilman … and Alexey Matveyevich Olovnikov…” Interestingly, these two scholars represent reciprocal extremes of theorizing in gerontology. Olovnikov in his “marginotomy theory” traced the causes of aging down to intracellular molecular changes, namely to the attrition of DNA ends and to the resulting telomere shortening. Dilman in his “elevation theory” traced the causes up to changes in the higher regulatory functions of the body, namely to the elevation of the threshold of sensitivity of the hypothalamus to homeostatic signals. It is also worth mentioning that both personalities epitomize a distinctive feature of Russian science tradition: “A broadly generalized interdisciplinary approach to scientific problems, with an intermingle of the elements of natural science, philosophy and culture, was always characteristic for the Russian school of thinking” [1].

Parallelisms between Blagosklonny’s and Dilman’s ideas have already drawn attention [2]. In the present essay, the genesis of these parallelisms will be addressed.

Vladimir Dilman was educated in internal medicine at the I.P. Pavlov 1st Medical Institute in Leningrad and started his research career there as an endocrinologist Figure 1. His candidate of medical sciences dissertation (equivalent to an MD), defended in 1955, was entitled “On the origin of climacteric and the role of age-associated changes in blood pressure, blood cholesterol and body weight”^1^. The founder of the Department of General Therapy, where this work was carried out, was Prof. G.F. Lang. He is characterized in [1] as follows: “This physician was the pioneer in formulation of the concepts for two most important diseases of modern elderly people: metabolic syndrome and, especially, hypertonic disease, which term and idea were coined by him (Lang, 1922; 1950).” As to Dilman’s tutor Prof. V.G. Baranov, who was a protégé of Lang, it is claimed that “As early as 1926, he was the first to formulate the principle of mandatory glycemia normalization, the correctness of which was confirmed many years later by the Diabetes Control and Complication Trial (DCCT). As far back as in 1932, he formulated the concept of relative and absolute insulin deficiency, which was introduced into all educational manuals on endocrinology. The classical theory of hyperinsulinemia in the early stages of type 2 diabetes mellitus and insulin resistance of peripheral tissues was also formulated by V.G. Baranov. Several decades ago, he developed a classification of diabetes mellitus, in which he combined the concepts of insulin-dependent and insulin-independent diabetes mellitus (diabetes mellitus types 1 and 2 in modern classification)” [1]. In view of what have been cited above, it is striking that Baranov is not even mentioned in publications on the history of diabetes research, such as [3, 4]. This almost certainly reflects the relative isolation of Russian (Soviet) science from the rest of the world, which was detrimental to both East and West. It is also important because age-associated hyperinsulinemia took central stage in those aspects of Dilman’s views on aging and carcinogenesis that, having been developed from Baranov’s ideas rather than picked up from abroad, were inherited by the hyperfunction concept of Blagosklonny.

**Figure 1:**
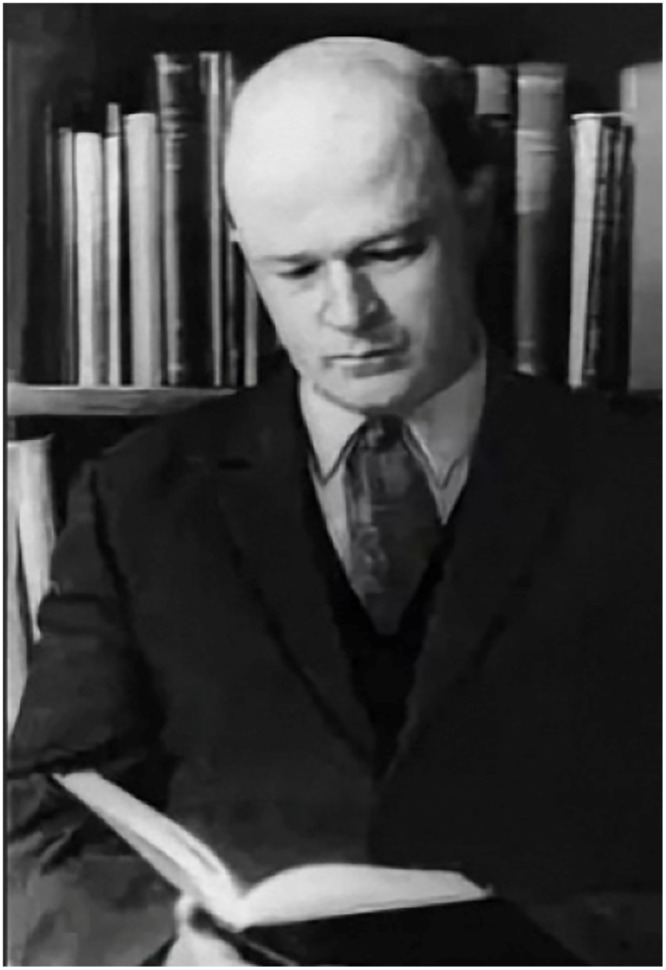
Professor V.M. Dilman

The principal conceptual aspects of Dilman’s dissertation were published under the title “Age-associated increase in the activity of some hypothalamic centers” in an institutional Russian journal in 1958 [5]. Ten years later, a monograph whose title in English is “Aging, Climacteric, and Cancer” was published by Dilman [6]. Soon after this, in 1971, his paper “Age-associated elevation of hypothalamic threshold to feedback control, and its role in development, aging, and disease” was published in "The Lancet" [7]. In the period of Cold War isolation, this was an outstanding accomplishment for a Soviet scholar. Due to it, Dilman gained worldwide recognition as an important contributor to the neuroendocrine variety of theories of aging [8–11]. Much consideration was also given to the following claim put forward in this paper: “More attention should be paid to phenformin, which eliminates compensatory hyperinsulinism and thereby reduces the cholesterol and triglyceride levels, and reduces excess body weight in aged patients. With this in view, phenformin should be investigated with regard to its ability to suppress the development not only of atherosclerosis, but also of tumour formation”.

The ability of this antidiabetic biguanide to inhibit carcinogenesis and to increase lifespan in rodents was demonstrated soon after (1974–1980) in a series of experiments performed in collaboration with Vladimir N. Anisimov in the Laboratory of Endocrinology (N.N. Petrov Research Institute of Oncology, Leningrad) headed by Dilman (reviewed in [12]). The reasoning behind these experiments was the “elevation theory of aging”, which derives aging-associated metabolic shifts from programmatic changes in the hypothalamus. Who could know then that in the 2010s it would be shown that the molecular biological effects of antidiabetic biguanides include mTOR inhibition in peripheral tissues (see [13]) and that mTOR would take center stage in the hyperfunction theory of aging developed by Blagosklonny? Notably, to check whether mTOR inhibition by rapamycin can produce anticancer and antiaging effects, Blagosklonny in 2010–2011, when already living in the USA, turned to collaboration with the same Anisimov at the same N.N. Petrov Research Institute of Oncology [14, 15]. The contribution of Anisimov, who by that time was President of the Gerontological Society of the Russian Academy of Sciences, to validating the ideas of both Dilman and Blagosklonny cannot be overestimated.

Dilman’s seminal paper in "The Lancet" [5] is remarkable not only in its contents but also in some concomitant respects. First, despite the Iron Curtain, several scores of papers published abroad were cited. Note that this was long before PubMed came to existence, not to mention WoS, Scopus, Google Scholar, and the online availability of the digital versions of scholarly papers. It is true that Soviet libraries were equipped exceptionally well. But it is also true that great motivation and perseverance were required then to trace relevant literature back and forward by carrying manual scores of weighty stacks of hardcopy journals from bookshelf to writing desk and back again. Second, most of what are currently regarded as indispensable components of the endocrine feedback implicated in metabolic control (adipokines, leptin, IL6, GLP, and GDF15 to name some) were unknown at that time. Third, in the section “Other theories of aging”, the biochemical theories, including the free-radical theory, which had been already reviewed as early as in 1961 [16], were not even mentioned, except in the remark that “other factors may exist in parallel and independently”. This lack of interest in molecular damage is noteworthy in view of the scrupulousness of Dilman’s approach to literature.

At this point, when discussing the relationship between Dilman’s and Blagosklonny’s ideas, it is instructive to compare the landscapes of aging-related scholarship during the periods when each came to prominence. As shown in Figure 2, the cumulative body of literature on aging in Dilman’s era was at least ten times smaller than in Blagosklonny’s. At that time, an exceptionally determined scholar could conceivably review the roughly two thousand papers published annually. Today, the volume of aging research has expanded to a level that makes it virtually impossible for any individual to stay fully informed. This growth reflects not only the overall proliferation of scientific publications but also a marked rise in the proportion devoted specifically to aging, particularly over the past decade.

**Figure 2:**
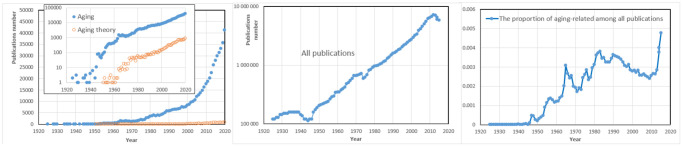
**Historical trends of the annual numbers of scholarly publications on aging.** Numerical estimates are obtained from PubMed. Left: Returns to queries “aging” and “aging theory” (a semilogarithmic plot is in the insert). Center: Semilogarithmic plot for all publications indexed in PubMed. Right: Proportion of aging-related publications among all indexed publications.

Before World War II, relatively few studies on aging were published. Clearly, any generalizations concerning aging could not be other than largely speculative then. The first monographs presenting relatively well-substantiated views began to appear in the 1950s (for example, Alex Comfort’s "The Biology of Senescence"). Their authors could claim then, and even two decades later, that they are comprehensively familiar with the available knowledge on the topic. Given the vast increase in publication output, it is no longer possible for anyone to keep pace with the entire field. Researchers can only choose particular studies they deem most relevant or insightful. Inevitably, however, such choices are shaped by conceptual frameworks and generalizations that originated in earlier times—when maintaining a comprehensive view of the evidence was still within reach. Among the early, largely speculative, generalizations about aging, those that combined sound logic, engaging style, and a more optimistic outlook tended to be the most persuasive. In this respect, Dilman was a true virtuoso. His enduring insistence that aging results not from wear and tear, deterioration, or loss, but rather from processes of enhancement, amplification, and overstimulation arising from a single underlying source, was both provocative and inspiring. The notion that one could counteract aging by inhibiting a single driving force, rather than attempting to repair a multitude of deficits, held an undeniable appeal. Particularly striking was Dilman’s emphasis on the increase in the hypothalamic sensitivity threshold—his so-called “elevation mechanism of aging”—rather than a decrease in hypothalamic sensitivity to homeostatic signals. This concept became the leitmotif of his writings: celebrated by newcomers to the field, yet often met with skepticism by established experts, e.g., [9].

Among such novices was the author of the present essay. Having gained a PhD in biochemistry and searching for a decent application point for my certified skills, I accidentally came across a publication authored by Dilman. I was immediately captivated by its arguments and struck by the realization that, after nearly a decade of studying biology, I remained woefully ignorant of the phenomenon of aging despite its evident, central importance to both academic biology and human life. Regrettably, the situation for students today is not much different.

The novice, whose dissertation was about the effects of experimental thyrotoxicosis on various dehydrogenases, then conceived that decreased thyroid functions in aged subjects would result in a decrease in the mitochondrial α-glycerol phosphate dehydrogenase, which oxidizes this substrate of triglyceride formation. Thus, with aging, the availability of this substrate must increase. That is what might cause age-associated hyperlipidemia, obesity, and their adverse consequences.

This hypothesis was all wrong, at the second glance. However, before giving it such a glance, I arranged a meeting with Dilman to get his opinion of my hypothesis. Predictably, he quickly rejected it, yet not because of its biochemical inconsistencies, but rather for reasons of principle: because a decrease (in thyroid function) was implied to be the primary driver of aging-associated metabolic changes. After a certain amount of initial mockery, I was invited to take part in developing a new idea of Dilman’s, that of metabolic immunodepression, and was handed a reprint of Dilman’s latest paper on this topic “Aging, metabolic immunodepression and carcinogenesis”, published in 1978 in the journal "Mechanisms of Ageing and Development" [17].

I was initially overwhelmed not only by the amount of information unfamiliar to me even after years of studying biology, but also the eloquence of its presentation. Needless to say, I accepted the challenge. The basic idea behind metabolic immunodepression still implied a sort of hyperfunction; in particular, hyperlipidemia was proposed to push cholesterol into lymphocytes making them less responsive to immunogenic stimuli. Moreover, this was proposed to be adaptive in that it helped avoid fetus rejection by the immune system during pregnancy, which is usually associated with hyperlipidemia. Nothing in a living body is for nothing, but processes may persist when nothing makes them still useful.

However, delving into details suggested that things were not so straightforward [18]. Cholesterol-rich low-density lipoproteins (LDL) do suppress lymphocyte responses to antigens, but do it by acting as whole particles rather than by pushing cholesterol into cell membranes. It appeared that LDL are not designed to suppress immunity during pregnancy; rather, their physicochemical properties, which make them able to be lipid carriers, unintendedly make them able also to block interactions between lymphocytes and antigens. For me this was a revelation, which eventually made me realize that similar futile tricks may be performed by other bodily constituents. In particular, catecholamines, suspected by Dilman to be involved in the elevation of the hypothalamic sensitivity threshold, feature chemical properties that make them, unlike all other neuromediators, toxic to neurons.

On the one hand, this idea initiated a series of publications on the significance for aging of excessive chemical reactivity of metabolites [19–23]. These examples of how one may find the ultimate causes of aging in one’s educational background (see below) could certainly not have been conceived had I not been infected by Dilman with an interest in gerontology.

On the other hand, such a dissident view could hardly be tolerated in a laboratory governed by the “law of deviation of homeostasis,” according to which the elevation of hypothalamic activity persists into adult life, continuing beyond its original purpose of guiding the body to maturity. In discussions of these issues, I pointed to organisms that undergo aging despite lacking a hypothalamus altogether, and to the necessity of integrating evolutionary perspectives into any comprehensive theory of aging. Misha Blagosklonny, who was then a rookie student at I.P. Pavlov 1st Medical Institute, was summoned by his father to take part in some of them. There were even attempts to come to a consensus (see sections VII and X in [24]), which however proved to be hopeless.

Vladimir Dilman was an unsurpassed master of supporting his theoretical constructions with scaffolds hastily assembled of very diverse materials. His immunological arguments were highly persuasive for endocrinologists and, at least initially, to biochemists, whereas his endocrinological arguments were praised by immunologists, and everyone was fascinated with his rhetoric and erudition including profound acquaintance with Russian literature. His favorite poet was Fyodor Tyutchev, and a rhyme he often quoted was:

**Table:** 

Priroda – sfinks, ee tem ona verney Svoim iskusom gubit cheloveka Chto, mozhet statsa, nikakoy ot veka Zagadki net ee ne bylo oo ney.	Nature's a sphinx, and through her test for man More surely brings about his tragic fall, In that, perhaps, she doesn't have at all A riddle, nor e'er did, since time began. https://lyricstranslate.com/en/priroda-sfinks-natures-sphinx.html

Among the lessons taught by apprenticeship in Dilman’s lab to me, then a snobbish graduate of an academic biochemical laboratory, finding himself immersed in a hot biomedical environment, one lesson relates to the understanding of complex phenomena, such as aging. When people with different educational backgrounds try to explain one thing with another, the other with yet another, and so on, they may eventually arrive at a point at which they feel that no further explanations are required. This point may be sensed intuitively, based on their routine experiences and influenced by the conceptual bents of their specific fields. Retroactively, this observation was found to be consistent with the “conceptual metaphor” theory [25]. Thus, in their explanations for aging, endocrinologists, e.g. Dilman, end with explanations in terms of higher neuroendocrine functions, chemists, e.g. Denham Harman, with free radicals [26], biochemists, e.g. I myself, with excessive reactivity of metabolites, immunologists, e.g. Roy Walford, with autoimmunity [27], whereas physicists may find satisfaction in explanations in terms of entropy increase.

Arguably, Dilman’s preoccupation, in conformance to his endocrinologist’s routine experience, with tracing the causes of age-related changes in the body to the hypothalamus, and treating the causes as a direct continuation of what occurs there during body development, hampered rather than enhanced the acceptance of his ideas (see e.g., comments by the eminent neuroendocrinologist Joseph Meites [9]), which he promoted by introducing arresting neologisms, such as hyperadaptosis, insulinization, and cancrophilia. Yet it is fascinating how much the attention paid by him to systems of interrelated changes, rather than to their individual components, helped him to anticipate several ways of thinking that are gaining increasing recognition today.

Obviously, local changes in the hypothalamus significantly affect the whole body via the endocrine system. Aging-related hypothalamic changes were discussed as avidly fifty years ago as they are in the current literature [28–31], however with little reference to the scholars, Dilman included, who long ago anticipated the importance of the systemic consequences of hypothalamic age-related changes.

A key question here is whether changes in hypothalamic function are programmed throughout the whole lifespan. They certainly are during development and, especially, upon reaching puberty. A further question is whether the developmental and aging-related changes are unidirectional. Dilman believed that most of them are. Even if this is so, are the causes of the unidirectional changes in hypothalamic responses to homeostatic inputs the same in development and aging? Much of what occurs in the hypothalamus during aging is now attributed to local inflammation [28]. Could similar biology really be implicated in the sexual maturation program? Could the deterioration of hypothalamic catecholaminergic systems that is caused by catecholamine toxicity [22] also be implicated in maturation?

While preparing this article, I came across a new publication [32] showing that the systemic hypoglycemic effect of metformin is mediated by inhibiting a small GTPase protein Rap1, specifically in the ventromedial hypothalamus. Could it be that the activity of this protein programmatically increases during development and continues increasing during aging according to the same program?

It is unquestionable that the way a body ages is determined by how it has been organized by the program of its development (or of complete metamorphosis in some cases, which may outnumber the cases of gradual development in the metazoan tree of life). Beyond this one could argue that aging takes place >within a result of the fulfilment of the developmental program, but not *as* the result of the program. According to this view (my own), developmental programs determine the conditions for aging to unfold but do not drive the unfolding thereof.

Whatever the causes of the age-related changes in the hypothalamus, which operate within one of the products of mammalian development, their consequences include systemic changes conducive to carcinogenesis. The current shift of attention from mutation accumulation in cells to changes in bodily conditions as a significant driver of the increasing incidence of oncological problems with aging is praised now as a “paradigm shift” [33]. However, the age-related changes in the “internal milieu of the organism”, as Dilman himself would call it, make the milieu increasingly similar to what he termed cancrophilia, which is commonly recognized now as the metabolic syndrome, the prevalence and severity of which increase with aging. Comparing the manifestations of metabolic syndrome [34] and cancrophilia [24] one cannot fail to recognize their high degree of equivalence.

The metabolic syndrome is associated with impaired glucose tolerance, excess body weight, and cardiovascular problems. Its association with oncological problems has attracted increased attention recently [35, 36], although their association with it (with cancrophilia in Dilman’s terminology) became apparent from studies conducted in Dilman’s laboratory as far back as the 1970–80s [24, 37]. Considerations put forward by Dilman on the commonality in the pathogenesis of neoplastic and atherosclerotic lesions remain relevant to this day [38, 39], although without due attribution.

An important factor by which cancrophilia can contribute to carcinogenesis, in Dilman’s views, is the suppression of cellular immunity resulting mainly from hyperlipidemia. The existence of this phenomenon, metabolic immunodepression (already described), has been confirmed in experiments and clinical studies using such tests as lymphocyte blast transformation in response to polyclonal mitogens, and slow-type skin hypersensitivity tests (reviewed in [18]). It should be admitted that 40 years ago, compared to the present, there was too little known about the organization of the immune system, including ignorance of the existence of even such cell types as natural killer cells and dendritic cells, to be able to formulate relations between anticancer immunity and systemic metabolism in terms currently adopted. However, Dilman was a pioneer in exploring the dependence of immunity on metabolic conditions. This topic is now attracting growing attention [40].

The key factor in the development of cancrophilia (cf. metabolic syndrome) was considered by Dilman to decreased glucose tolerance. Correlations between hyperglycemia and cancer risk have been found in epidemiological studies (reviewed in [41]); however, the mechanistic basis of this correlation may be not only hyperglycemia-associated hyperinsulinemia but also glucose itself, its availability to cells and the exposure of cells to nonenzymatic glycation by its linear form. Nevertheless, the usability of drugs active against hyperglycemia, such as antidiabetic biguanides, for preventing and treating cancer was anticipated by Dilman long before the first epidemiological evidence of reduced cancer incidence among metformin users was published (reviewed in [12]). The recent paper showing that one-hour post-load blood glucose level is the best correlate of mortality rates attributed to atherosclerosis and cancer [42] would be regarded by Dilman as a splendid icing on his cake given its good fit with his cancrophilia model. However, there are no references to his work in this publication either. The same is true for the recent “discoveries” of cancer associations with insulin resistance [43] and with hypertriglyceridemia [44], both of which were demonstrated in Dilman’s laboratory 40 years ago [45].

Here, I would like to cite the Editorial to the Special Issue of "The Gerontologist" journal entitled “Remembering Our Roots” [46]: “Science moves forward as scholars build on the work of those who preceded them. Students are taught that familiarity with literature allows an investigator to avoid needless repetition of work that has been done before and to contribute something new. Citations to earlier work provide support for the validity of arguments and avenues for interested readers to pursue. Against this fundamental principle of science lies the daunting reality of most publications, "The Gerontologist" included, whose rigid word counts must be adhered to, and whose concerns about impact factors favor citation of recent publications over those of older and even classic works. A common, highly undesired result is that we as a discipline forget our past. We forget our forefathers and foremothers, and the fundamental ideas that shaped our discipline are relegated to the back burner.”

Actually, much of the geroscience agenda was anticipated by Dilman since; while tracing the causes of age-related hyperglycemia to the elevation of the threshold of sensitivity of the hypothalamus to glucose, he considered this as an aspect of the generalized elevation of the threshold of hypothalamic sensitivity to homeostatic signals. The latter is necessary to increase the power (in the sense not of higher accuracy, but higher amounts of body components to deal with) of the homeostatic systems implicated in reproduction, adaptation and resource handling. Increases in this power, which are required for development, were thought by him not to stop after its completion but to continue further, thus eventually causing aging and acting as its pacemaker. This leads to the main diseases of aging and associated deaths faster than stochastic damage can do. Therefore, to decelerate the increase in hypothalamic sensitivity threshold is to decelerate aging and the development of the diseases associated with it. All being that easy, it is no wonder that Nature “perhaps, doesn't have at all a riddle, nor e'er did, since time began”.

Having been brought by the intergenerational transfer of ideas to the molecular biological level, this approach eventually yielded claims (which are questionable in my view [23, 47, 48]) found in the titles of publications by Blagosklonny, such as “M(o)TOR of aging: mTOR as a universal molecular hypothalamus” [49], “Growth and aging: a common molecular mechanism” [50], and “Disease or not, aging is easily treatable” [51]. However, is it not true that provoking questions rather than suggesting allegedly ultimate answers is the principal driver of scientific progress to which Vladimir Dilman and Misha Blagosklonny contributed so much? And, more than that, they did it their way.

After all, as Dilman’s favorite poet once said:

**Table:** 

Nam ne dano predugadatt’ Kak slovo nashe otzovetsa...	We are not given to foretell How words we say will some time echo …

## Footnotes

1 In the Wikipedia article at https://en.wikipedia.org/wiki/Timeline_of_aging_research, the following may be found: “1954 Vladimir Dilman formulated the hypothesis of aging that at first become known only in the USSR, as the elevation hypothesis. In 1968 it took the form and became known as the neuroendocrine theory of aging”. Notably, this development is listed between “1952 Peter Medawar proposed the mutation accumulation theory to explain how the aging process could have evolved” and “1956 Denham Harman proposed the free-radical theory of aging and demonstrated that free radical reactions contribute to the degradation of biological systems” followed by “1957 George Williams proposed the antagonistic pleiotropy hypothesis for the explanation of the emergence of aging”.
